# De novo variation in *EP300* gene cause Rubinstein-Taybi syndrome 2 in a Chinese family with severe early-onset high myopia

**DOI:** 10.1186/s12920-023-01516-9

**Published:** 2023-04-21

**Authors:** Xiaoyu Huang, Xue Rui, Shuang Zhang, Xiaolong Qi, Weining Rong, Xunlun Sheng

**Affiliations:** 1 Clinical Medical College, Ningxia Medical University, No.692 Shengli Street, Xingqing District, Yinchuan, China; 2 Eye Hospital, School of Optometry and Ophthalmology, Wenzhou Medical University, 270 Xueyuan Road, Wenzhou, Zhejiang 325027 China; 3 Ningxia Eye Hospital, People’s Hospital of Ningxia Hui Autonomous Region, Third Clinical Medical College of Ningxia Medical University, 936 Huanghe East Road, Jinfeng District, Yinchuan, 750004 China; 4 Gansu Aier Ophthalmology and Optometry Hospital, 1228-437, Guazhou Road, Qilihe District, Lanzhou City, Gansu 730050 China; 5 Department of Ophthalmology, Ningxia Eye Hospital, People’s Hospital of Ningxia Hui Autonomous Region, Third Clinical Medical College of Ningxia Medical University, 936 Huanghe East Road, Jinfeng District, Yinchuan, 750004 China; 6 Gansu Aier Ophthalmology and Optometry Hospital, 1228 Guazhou Road, Qilihe Qu, Lanzhou, 730050 China

**Keywords:** *EP300* gene, Early onset high myopia, Phenotype, Rubinstein-Taybi syndrome 2

## Abstract

**Background:**

Rubinstein-Taybi syndrome (RSTS) is characterized by distinctive facial features, broad and often angulated thumbs and halluces, short stature, and moderate-to-severe intellectual disability, classified into two types RSTS1 (*CREBBP*-RSTS) and RSTS2 (*EP300*-RSTS). More often, the clinical features are inconclusive and the diagnosis of RSTS is established in a proband with identification of a heterozygous pathogenic variant in *CREBBP* or *EP300* to confirm the diagnosis.

**Methods:**

In this study, to describe an association between the clinical phenotype and the genotype of a RSTS2 patient who was initially diagnosed with severe early-onset high myopia (eoHM) from a healthy Chinese family, we tested the proband of this family by whole exome sequencing (WES) and further verified among other family members by Sanger sequencing. Real-time quantitative PCR was used to detect differences in the relative mRNA expression of candidate genes available in the proband and family members. Comprehensive ophthalmic tests as well as other systemic examinations were also performed on participants with various genotypes.

**Results:**

Whole-exome sequencing revealed that the proband carried the heterozygous frameshift deletion variant c.3714_3715del (p.Leu1239Glyfs*3) in the *EP300* gene, which was not carried by the normal parents and young sister as verified by Sanger sequencing, indicating that the variant was *de novo*. Real-time quantitative PCR showed that the mRNA expression of *EP300* gene was lower in the proband than in other normal family members, indicating that such a variant caused an effect on gene function at the mRNA expression level. The variant was classified as pathogenic as assessed by the interpretation principles of HGMD sequence variants and ACMG guidelines. According to ACMG guidelines, the heterozygous frameshift deletion variant c.3714_3715del (p.Leu1239Glyfs*3) in the *EP300* gene was more likely the pathogenic variant of this family with RSTS2.

**Conclusions:**

Therefore, in this paper, we first report *de novo* heterozygous variation in *EP300* causing eoHM-RSTS. Our study extends the genotypic spectrums for *EP300*-RSTS and better assists physicians in predicting, diagnosis, genetic counseling, eugenics guidance and gene therapy for *EP300*-RSTS.

## Background

Rubinstein-Taybi syndrome (RSTS; MIM 180,849 and 613,684) is a rare autosomal dominant disorder [1] with an incidence of 1:100,000 to 1:125,000 in the population [2, 3]. RSTS was first identified and reported by pediatrician J. H. Rubinstein and radiologist H. Taybi in 1963 [4]. The main clinical features are distinctive facial features, broad and often angulated thumbs and toes, short stature and mental retardation. Other systemic manifestations include obesity (BMI:24.4 kg/m2), decreased social interaction, instability of mood, and more limited speech. Although RSTS is inherited in an autosomal dominant manner, approximately 99% of patients represent simplex cases as the result of pathogenic variants occurring *de novo* in the family. For example, only one affected child was diagnosed with RSTS but the parents were healthy [5]. Thus, only a few familial cases have been reported so far. The pathogenesis of RSTS was found to be associated with two highly evolutionarily conserved genes, and depending on the pathogenic genes, RSTS can be classified into RSTS1 (RSTS1, NM_004380.3) due to *CREBBP* gene variants and RSTS2 (RSTS2, NM_001429.4) due to *EP300* gene variant. Approximately 60% of RSTS is caused by *CREBBP* gene variants, 8–10% of RSTS is caused by *EP300* gene variants [6].

The growth rate of RSTS patients is generally unaffected during fetal life, and in the months after birth, patients rapidly lose height, weight, and head circumference below the fifth percentile, and adult patients are usually short in stature and are beginning to appear obese during adolescence and adulthood [7]. As a multi-system disease, some RSTS patients may have combined ocular, cardiac, renal, and genital abnormalities [8]. Among the ocular abnormalities, strabismus is the most common, accounting for approximately 61–79%, refractive error (41–56%), lacrimal duct obstruction (38–47%), glaucoma, monocular/bilateral iris, retinal, optic nerve defects, and cataracts are also common [9, 10]. As compared to patients with the *CREBBP* gene variant, patients carrying the *EP300* gene variant have a relatively milder clinical phenotype, particularly milder skeletal abnormalities of the thumbs and toes and less severe intellectual disability [11, 12]. Although more than 1000 cases of RSTS have been reported in the literature and a variety of clinical phenotypes have been described including some rare signs and symptoms [13], which means the clinical phenotype of RSTS is complex and diverse, and the relationship between genotype and clinical phenotype is unclear. Therefore, it is often difficult or impossible to make an accurate clinical diagnosis of RSTS based solely on clinical symptoms, signs, and some ancillary examinations, relying on the physician’s assessment of the patient’s psychological, physical, and morphological abnormalities.

Due to the heterogeneity of the clinical manifestations of RSTS and the fact that no comprehensive and uniform clinical criteria have been worked out, most cases of RSTS need to be diagnosed definitively by genetic testing. The advent of whole-exome sequencing (WES) technology has improved the level of accurate diagnosis of such diseases and changed the process of defining the phenotypic spectrum of the disease. The phenotypic spectrum of known Mendelian diseases has been expanded accordingly.

Here, we report a patient who presented to Ningxia Eye Hospital with early-onset high myopia (eoHM) from a healthy Chinese family, in which some phenotypes resembled RSTS2. The WES was performed to detect genetic variants, and the genotypic and clinical phenotypic characteristics of RSTS-related eye diseases were discussed, providing reliable molecular diagnosis for the eoHM-RSTS2 and helping clinicians to improve their awareness of the disease.

## Materials and methods

### Clinical observations and analysis

This study was performed according to the Declaration of Helsinki and was approved and reviewed by the Ethics Committee on Human Research at People Hospital of Ningxia Hui Autonomous Region[Approval No.20,190,909]. Informed consent was obtained from all the participants including parents and children.

The proband (II-1) is an 11-year-old boy from a healthy unrelated couple (I-1, I-2). He has a younger sister (II-2). All individuals in this family were recruited for both clinical and genetic tests. Comprehensive ophthalmic examinations were performed on subjects, including Best-Corrected Visual Acuity (BCVA), slit lamp microscopic examination, intraocular pressure (IOP), axial Length (AL), corneal curvature (CC), anterior chamber depth (ACD), visual fields (VF), full-field electroretinography (ffERG), optical coherence tomography (OCT). In addition, the facial appearance photo and hands & feet appearance photo were taken in the proband.

AL, CC, and ACD were recorded by IOL Master Optical Biometry (IOL Master 500, Carl Zeiss Meditec AG). Images of color fundus photography were captured from fundus photography analyzer (TRC-NW300, TOPCON, Japan). The visual field testing was performed with Humphrey Field Analyzer (750i Carl Zeiss Meditec, USA) 30–2 program, inspection range: 30 degrees with 76 points grid, background brightness: 31.5asb. ffERG was performed using corneal ERG jet contact lens electrodes (RetiPort ERG system; Roland Consult, Wiesbaden, Germany), according to the International Society of Clinical Electrophysiology of Vision (ISCEV) standards (21, 22). Cirrus high definition optical coherence tomography (HD-OCT4000, Carl Zeiss Meditec, USA) was performed as Macular cube 512 × 128 HD 5 line scans across the foveal center. Peripheral venous blood samples (3 mL) were collected from all individuals of the family for genomic DNA isolation.

### Whole exome sequencing

Whole-genome exome capture was performed using Agilent SureSelect exome capture kit, and high-throughput sequencer (Illumina) was performed to a depth of 100 ×. The raw sequencing data were processed by Illumina base calling Software 1.7 and subsequently compared with the NCBI human genomic DNA reference sequence (NCBI build 37.1). Single nucleotide variation (SNV) related information was analyzed by SOAP software (http://soap.genomics.org.cn), and the information related to inserts and deletions (Indel) was analyzed by BWA software (bio-bwa.sourceforge.net/) to obtain all the variants occurring in the DNA sequences in the samples. Quality control of sequencing data was performed subject to sequencing depth, the ratio of the number of variants reads measured to the total number of reads at the locus, whether it was in the high GC region, whether it was in the repetitive sequence region, and whether it was in the poly region. Database frequencies such as 1KG and gnomAD were screened for predictions using over 20 bioinformatics software to discern the relationship between the gene in which the variant was located and the disease. High-frequency variant loci with minimum allele frequency (MAF) > 1% were screened from the database (db135). Variants that did not affect the structure and function of the protein were screened out. After the stepwise screening, the number of variants shared by all probands in the family was screened out, and then variants unavailable with the diseased relatives in the family were screened to obtain the candidate pathogenic gene variants. Sanger validation was used to exclude false positives for candidate pathogenic genetic variants, while the presence of co-segregation was verified in normal family members.

### In silico analysis

The target variant loci were queried using database tools such as HGMD (human gene mutation database) and dbSNP (https://www.ncbi.nlm.nih.gov/snp/) to see if they were reported pathogenic variants in HGMD and to see if they had been included. In the case of novel or *de novo* variants that have not been reported, the variants were assessed for pathogenicity according to the Standards and Guidelines for Interpretation of Sequence Variants issued by the American College of Medical Genetics and Genomics (ACMG) in 2015. These ACMG Standards and Guidelines recommend the use of specific standard terminology—“pathogenic”, “likely pathogenic”, “uncertain significance”, “likely benign” and “benign”—to describe variants identified in genes that cause Mendelian disorders. In these five categories of classifying variants, the “Pathogenic” is classified as 1 and the “likely pathogenic”is classified as 2, “uncertain significance” is not considered a confirmed diagnosis.

MAF less than 0.005 was used as the criteria to exclude benign variants by reference to the databases for East Asian populations Allele frequencies available with 1000 Genomes Project (1000G, http://browser.1000genomes.org) and Exome Aggregation Consortium (https://gnomad.broadinstitute.org/). Such prediction software as Polyphen2(http://genetics.bwh.harvard.edu/pph2),SIFT (https://grch37.ensembl.org/Tools/VEP), PROVEAN (http://provean.jcvi.org/index.php) and Mutation Taster (http://www.mutationtaster.org) were used for pathogenicity prediction. Variants were classified as clinically unclear when at least 1 prediction had a benign outcome or when there was insufficient evidence of pathogenicity. When all predictions were pathogenic, variants were classified as likely pathogenic in combination with other evidence. Frameshift variants, nonsense variants, and variants with experimental evidence of loss of protein function were classified as pathogenic variants. The online analysis tool Multalin (http://sacs.ucsf.edu/cgi- bin/multalin.py) was used for the conservativeness analysis of the variant loci. Co-segregation analysis was performed using Sanger sequencing in the patient’s family members.

### Analysis of relative mRNA expression in candidate gene

The relative expression of *EP300* gene mRNA in patients and control samples (patient’s family members) was detected by RT-qPCR. RNA was extracted from blood samples using the PAXgene Blood RNA Kit (Qiagen #762,174), followed by determination of RNA concentration and quality using a Qubit® 3.0 fluorometer (Life Technologies, USA) and Nanodrop One spectrophotometer (Thermo Fisher Scientific Inc, USA), and assessment of total RNA integrity using an Agilent 2100 Bioanalyzer (Agilent Technologies Inc, USA). 1 µg of RNA was taken for reverse transcription using the Hifair® II 1st Strand cDNA Synthesis Kit (gDNA digester plus) (Yeason Biotech, 11121ES50). Intron-crossing primers for the *EP300* gene were designed. Forward primer-CAGTATGTCGATGATATTTGGCTT and reverse primer-CAACTTTCTGCCACAACAGTATC.The RT-qPCR reaction system was prepared according to the NovoStart® SYBR qPCR SuperMix Plus kit. The 20 µL system contained 10 µL of 2×NovoStart® SYBR qPCR SuperMix Plus, 0.5 µL of forward and reverse primers each, 1 µL of cDNA (10 ng/µL), and 8 µL of water. A program of initial denaturation (95 °C for 1 min) and 40 cycles of amplification (95 °C for 20 s, 60 °C for 20 s) was performed on a Roche Light Cycler II 480 real-time fluorescent quantitative PCR instrument with 3 replicates set for each reaction. The 2-ΔΔCt method was used to calculate the relative mRNA expression of *EP300* gene in the patient and control samples (patient’s family members), which was normalized by the Ct value of the internal reference GAPDH gene.

## Results

### Medical history and external characteristics of the patient in this family

**Fig. 1:**
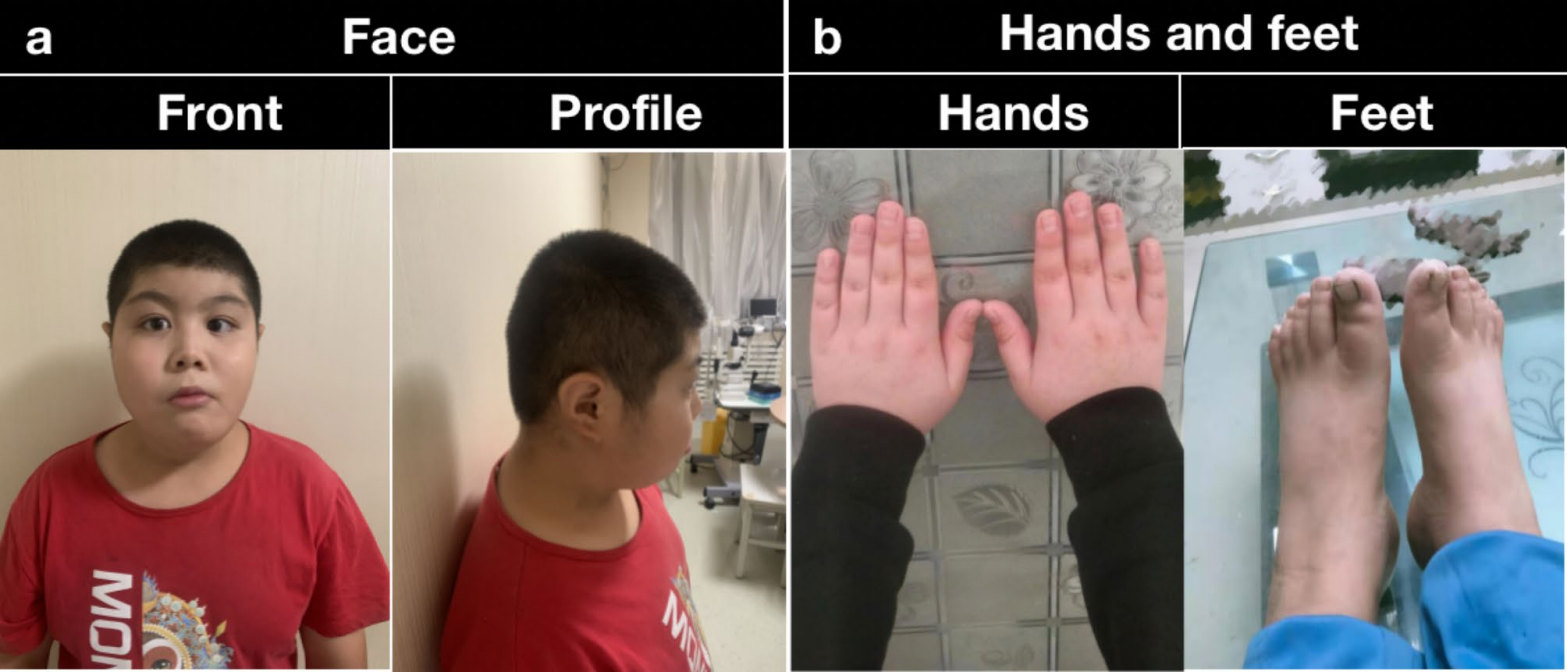
Photographs of the proband. (**a**) Photos of the face: low facial hairline (< 5 cm), high arched eyebrows, mildly downward palpebral fissures, long eyelashes, and convex nasal ridge with low-hanging columella. (**b**) Photos of hands and feet: slightly broad but not angulated thumbs and toes

The proband, a male, age of 11, visited Ningxia Eye Hospital due to blurred vision in both eyes for 6 years. Medical records of previous visits showed: the first visit to the ophthalmology department was made at the age of 5 years old with a diagnosis of high myopia (Table 1). The Peabody Picture Vocabulary Test suggested mild mental retardation. From the age of 1 to 7, he had frequent pneumonia and had no feeding difficulties. Parents denied consanguineous marriage, the mother had 2 pregnancies and 2 births, and no one in the family had clinical symptoms similar to the patient. Birth history: The child patient was delivered at full term by normal delivery, weighing 2750 g at birth (< 3300 g). He is 11 years old now, 150 cm in height (the middle level of height in normal 11-year-old Chinese children), and 55 kg in weight (BMI:24.4 kg/m2) (weight exceeded 97% of his peers). Facial manifestations: low facial hairline 3.5 cm (< 5 cm), arched eyebrows, long eyelashes, downward sloping palpebral fissures, and drooping nasal columella, and he also has wider and thicker thumbs/toes (Fig. 1). He has poor academic performance, does not communicate with his classmates, has no friends, and sometimes short-tempered. The child patient was found by his parents that his language skills had deteriorated in recent years and his speech was stuttering.

**Table 1:** Refraction and best corrected visual acuity(BCVA) of the proband in recent 6 years

Age(year)	OD		OS	
	BCVA	Refraction	BCVA	Refraction
5	0.25	-9.00DS/-3.25DC*175°	0.1	-9.50DS/-2.75DC*175°
6	0.3	-10.00DS/-3.50DC*175°	0.12	-10.00DS/-3.50DC*170°
7	0.5	-10.00DS/-3.00DC*5°	0.3	-10.50DS/-3.00DC*180°
8	0.6	-10.25DS/-3.00DC*5°	0.3^−^	-10.50DS/-3.00DC*175°
10	0.6^−^	-11.00DS/-2.75DC*13°	0.3^−^	-12.00DS/-2.50DC*180°
11	0.6^+^	-11.25DS/-2.50DC*10°	0.3	-12.25DS/-2.75DC*5°

### Refraction and fundus characteristics of the patient in this family

**Fig. 2:**
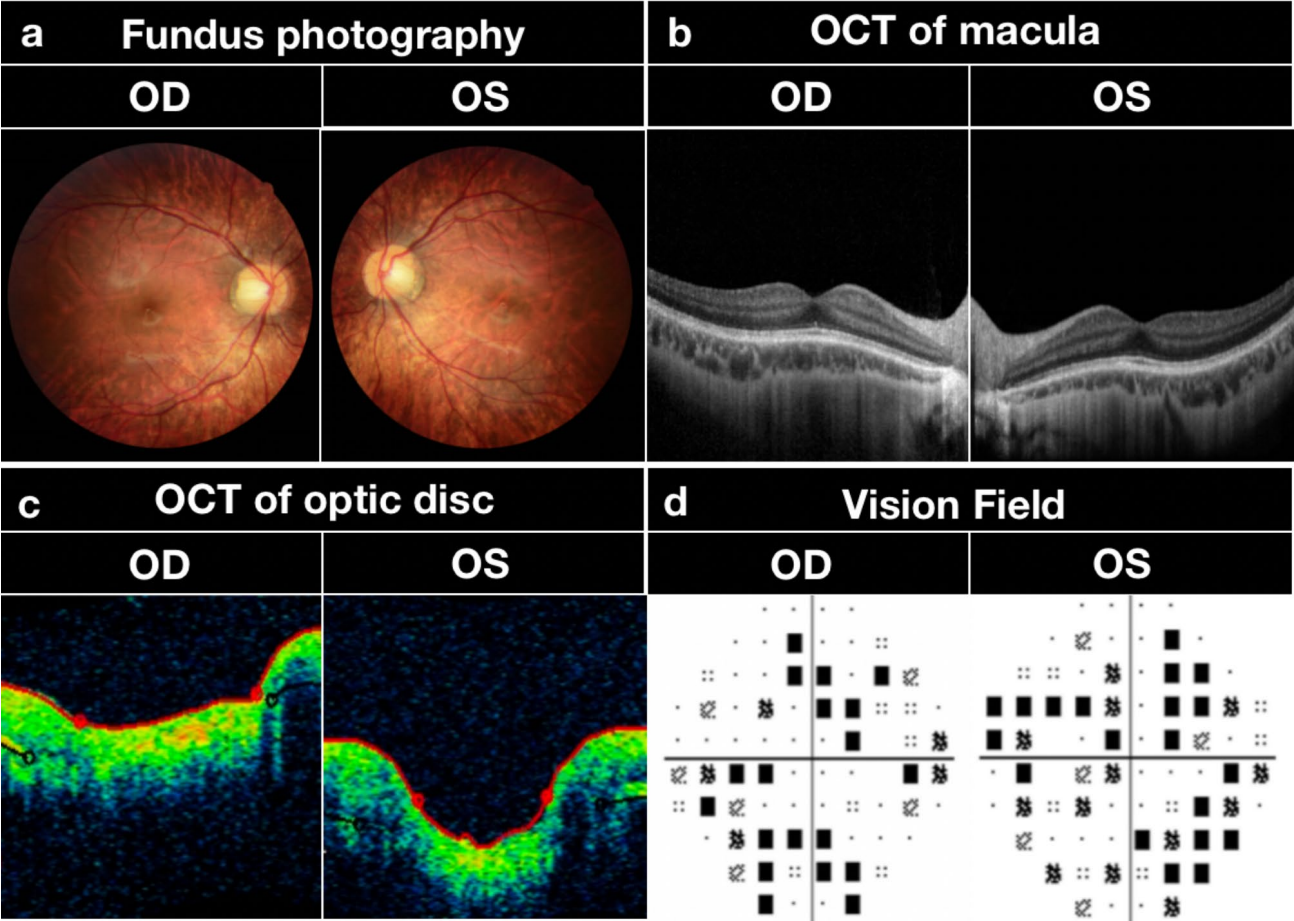
Fundus examination of the proband. (**a**) Fundus of both eyes: Fundus photography shows a diffuse chorioretinal atrophy and tessellation. (**b**) Optical Coherence tomography (OCT) of macula: no significant abnormalities. (**c**) OCT of optic disc: no significant abnormalities. (**d**) Vision Field: multiple central and peripheral scotomas

Ophthalmic examination showed BCVA: 0.6+(-11.25DS/-2.50DC*10°) in the right eye and 0.3 (-12.25DS/-2.75DC*5°) in the left eye. IOP: 11 mmHg for the right eye and 12 mmHg for the left eye. Synoptophore examination: simultaneous vision without a coincident point, objective angle + 12°for naked eyes, objective angle + 10°under wearing glasses. Irrigation of lacrimal passages was unobstructed in both eyes. Axial length: 26.59 mm in the right eye and 26.78 mm in the left eye. Corneal curvature: 42.00 D in both eyes. Anterior chamber depth: 3.48 mm in the right eye and 3.57 mm in the left eye. Fundus photography showed diffuse retinal atrophy and early stage of myopic maculopathy (tessellation), which presented well-defined choroidal vessels that can be observed clearly around the fovea as well as around the arcade vessels in both eyes. The OCT examination showed no significant abnormalities in macula and optic disc. The visual field defects in the center and periphery of both eyes and ERG showed no significant abnormalities (Fig. 2).

### Genetic test results

**Fig. 3:**
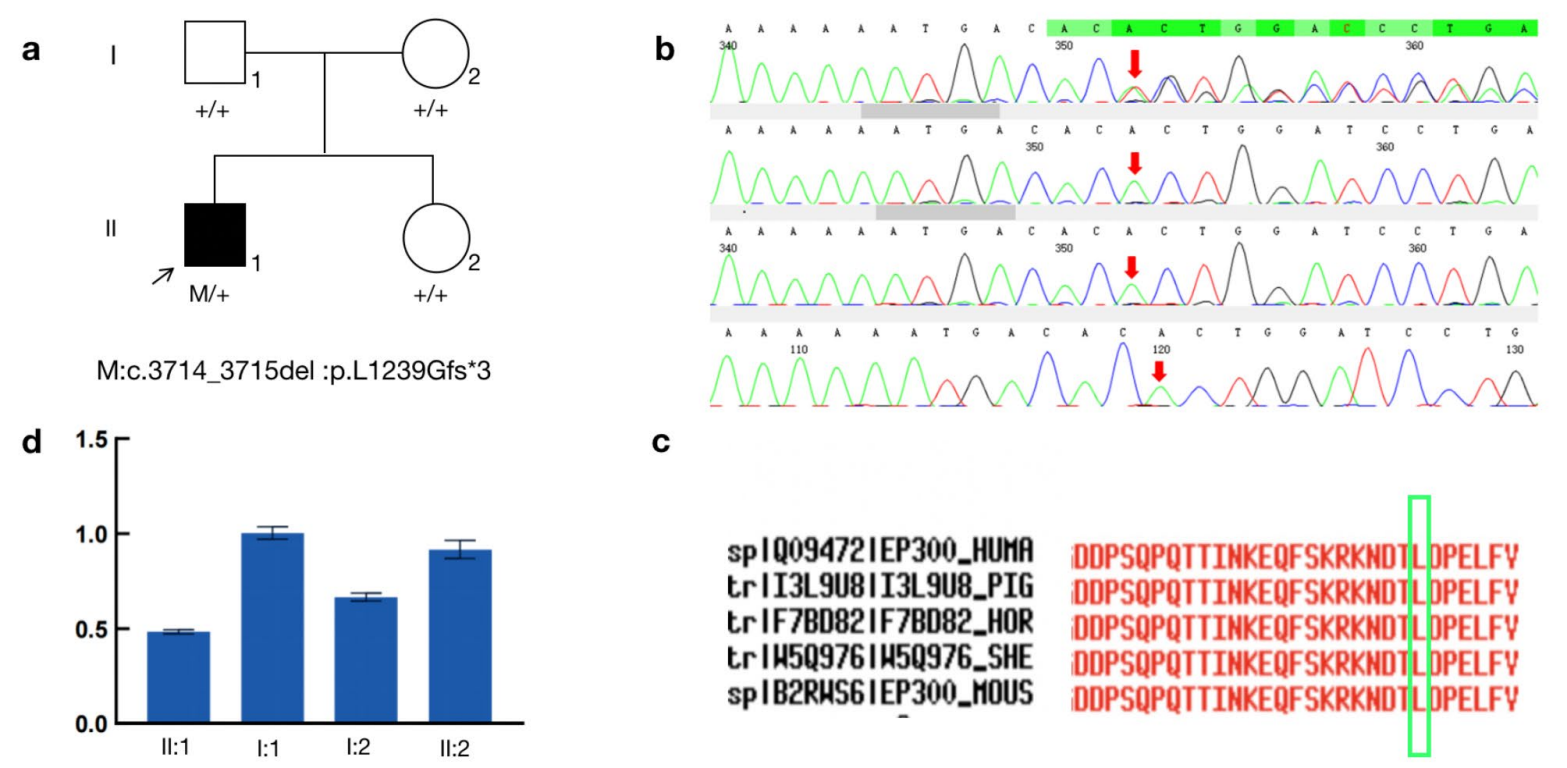
Sequence analysis and identification of the novel mutations of *EP300* in the affected family with autosomal-dominant RSTS. (**a**) Pedigree of the family. The filled black symbols represent the affected members and the arrow denotes the proband. (**b**) Sequence chromatograms of identified mutations. (**c**) The homology of amino acid sequences between human *EP300* and other species. The amino acid at position 1239 (Leucine, L1239) is highly conserved among species. (**d**) Relative gene expression levels of *EP300* in the affected family with autosomal-dominant RSTS:showing relative gene expression levels of *EP300* of II-1(0.49) is lower than I-1(1.04),I-2(0.69) and II-2(0.97) and P value (II-1 : I-1) = 0.0011, P value (II-1 : I-2) = 0.0043, P value (II-1 : II-2) = 0.0036

**Fig. 4:**
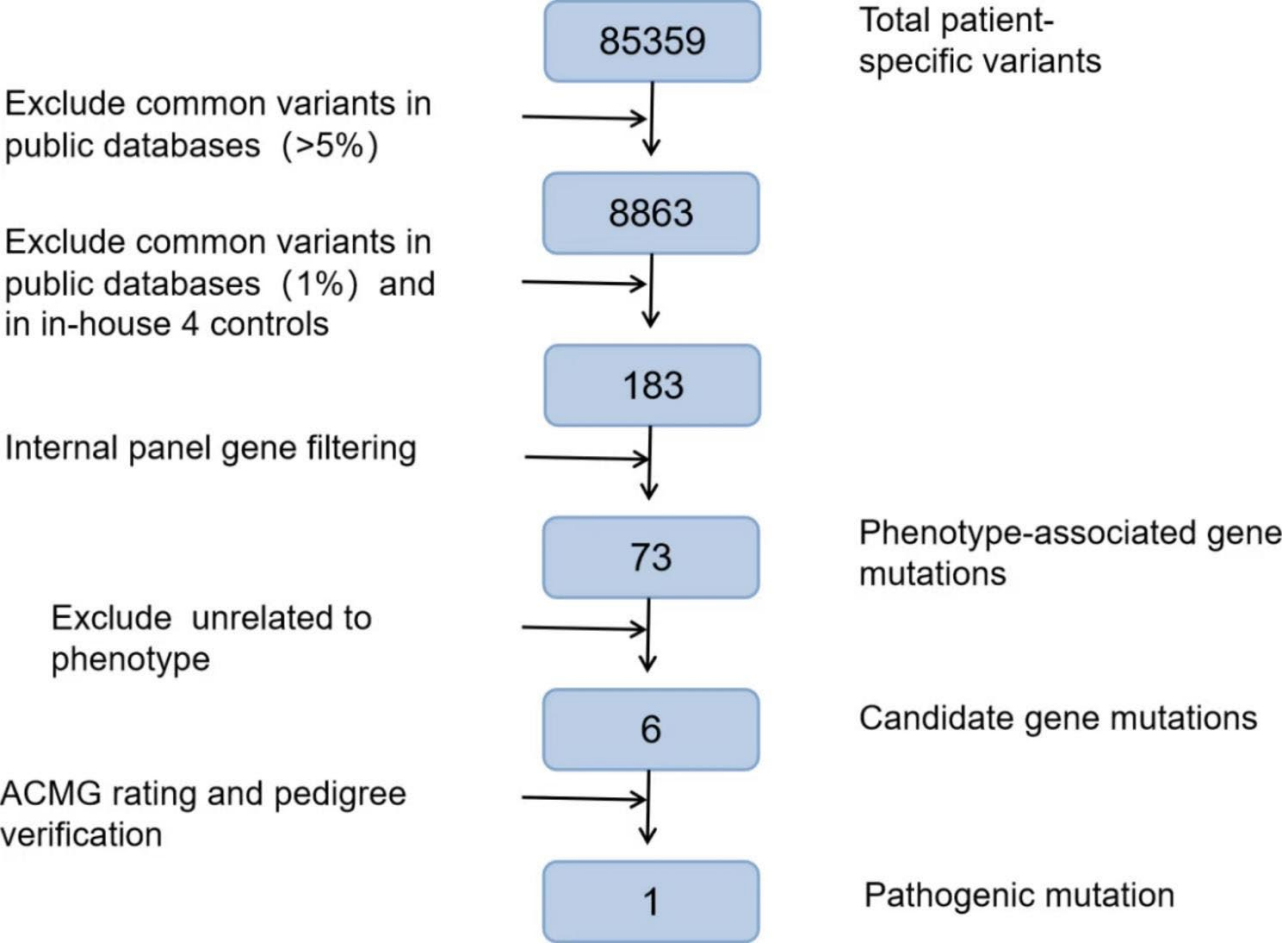
Work flow of WES analysis in this study. One variant in *EP300* was selected out of 6 candidate variants based on the scores of mathematical predictions of mutational impact on proteins and its pathological impact as well. c.3714_3715del, which results in following amino acid substitution: (p.Leu1239Glyfs*3)

To find the cause of the disease, we performed the WES of the eoHM patient (II-1) in this study and found the heterozygous nonsense variant in *EP300* NM_ 001429.4:exon21:c.3714_3715del(p.Leu1239Glyfs*3) (Fig. 3ab). Further verified in other family members (I-1, I-2, and II-2) by Sanger sequencing showed the c.3714_3715del(p.Leu1239Glyfs*3) was not carried by the normal parents and younger sister, indicating it is a *de novo* nonsense variant(PS2_strong). The frameshift variant has not been previously reported and was also not detected in the East Asian Population Database (ExAC_ EAS) (PM2_Moderate). The deletion of 2 nucleotides resulted in a substitution of leucine to glycine at codon 1239 followed by a frameshift, a substitution of aspartic acid to serine at codon 1240, and a premature stop codon (PTC) at position 1241(c.3719–3721) was created. The synthesis of the polypeptide chain was terminated in advance and truncated protein was produced accordingly. The truncate variants would result in the premature termination of polypeptide chain synthesis, and most of the proteins produced were inactive or lost their normal function (PVS1_Very Strong). Nonsense-mediated mRNA decay (NMD) is expected to eliminate *EP300* protein expression (including truncated polypeptides) from mutant alleles. Besides, the arginine at position 1239 was highly conserved among different species by proteomic conservation analysis (Fig. 3c), indicating that the nonsense variation at this site is more likely to affect the function of *EP300* protein. Taken together, this *de novo* nonsense variation in *EP300* is more likely to cause eoHM-RSTS2 by affecting the function of *EP300* protein(Pathogeni, Class1)(Fig. 4). Therefore, we used RT-qPCR to detect the expression of *EP300* gene transcript levels, i.e. relative expression of mRNA, in the proband and normal family members. The results showed that the mRNA expression of *EP300* gene available with the samples I-1(1.04)、I-2(0.69), II-2(0.97)of the proband (II-1) (0.49) was lower than that in the samples I-1 (1.04), I-2 (0.69), and II-2 (0.97) of the normal family members which P value (II-1 : I-1) = 0.0011, P value (II-1 : I-2) = 0.0043, P value (II-1 : II-2) = 0.0036(Fig. 3d), indicating that most of the mutated transcripts were eliminated before translation due to nonsense-mediated mRNA decay.

### Some literature review

A search of the previously reported relevant literature showed that pathogenic variants in the *EP300* gene resulted in a complex and diverse clinical phenotype. The Ocular phenotype of most of the previously reported cases was strabismus, refraction, and nasolacrimal duct obstruction. P300 (*EP300* binding protein) (22q13.2) is often expressed in the heart, nerve, kidney, lens, cornea, eyelid and photoreceptor cells. As histone acetyltransferase (HAT), P300 plays an important role in cell proliferation and differentiation through chromatin remodeling and regulating transcription [14]. The clinical manifestations of AS are diverse and have individual differences, as shown in Table 2.

**Table 2:** The manifestations of RSTS between the literature and the patient presented in this paper

Manifestations		RSTS	Patient	Literatures
**Eye**				
Refraction		+	+	[1, 4]
Strabismus		+	+	[1, 4]
Ptosis		+	-	[1, 4]
Nasolacrimal duct obstruction		+	-	[1, 9, 10]
Cataracts		+	-	[1, 4, 10]
Coloboma		+	-	[1, 8]
Nystagmus		+	-	[1]
Glaucoma		+	-	[1]
Corneal abnormalities		+	-	[4, 8]
Retinal abnormalities		+	-	[10]
**Face**				
Microcephaly		+	+	[6, 11, 12, 16]
Arched eyebrows		+	+	[1, 4, 6]
Down-slanting palpebral fissures		+	Mild	[1, 6, 11, 16]
Long eyelashes		+	+	[6]
Low-hanging columella		+	+	[1, 6, 11, 16]
High palate		+	-	[1, 4, 6]
Grimacing smile		+	-	[1, 6]
**System**				
Obesity		+	+	[1, 6]
Intellectual disability		+	+	[1, 4, 6, 12]
Broad and angulated thumbs/halluces		+	Mild	[1, 4, 6, 8, 11, 12]
Congenital heart disease		+	-	[1, 6, 12, 16]
Pneumonia		+	+	[1, 4, 8]
Undescended testicles		+	-	[1, 4, 8, 12]
Constipation		+	+	[1, 4]
Keloids		+	-	[1, 6, 29]
Decreased social interaction		+	+	[1]
Speech difficulties		+	+	[1, 12]
Instability of mood		+	+	[4]

RSTS : Rubinstein-Taybi syndrome

## Discussion

Rubinstein-Taybi syndrome is a rare genetic disorder characterized by postnatal growth retardation, moderate to severe intellectual disability, and a variety of characteristic malformations. Despite the prominent facial features and broad thumbs and hallux (toes), the diagnosis of RSTS is challenging due to the high phenotypic and genotypic variability [13].

*EP300* is located on chromosome 22q13.2 and contains 31 exons that encode the E1A-1-binding protein p30, consisting of 2415 amino acids [15]. *EP300* gene encodes paralogs acting as lysine acetyltransferase involved in transcriptional regulation and chromatin remodeling plays a key role in neuronal plasticity and cognition [16]. 118 variants in *EP300* gene have been reported so far [17, 18]. The mutational spectrum includes 84.7% point mutations of which 69.5% are truncating mutations, 5.1% splicing mutations, and 13.6% missense mutations for 11.8% large rearrangements [17, 18]. Like *CREBBP*, there are no hot spot mutations in *EP300* and only four pathogenic variants were reported more than twice in the database: three in the catalytic domain and one in exon 2 [17, 18].

In our case, the nonsense variant c.3714_3715del (p.Leu1239Glyfs*3) in *EP300* was detected in the proband, and neither the phenotypically normal parents nor the sister in the family carried such a variant, indicating that it was a *de novo* variant. RSTS is inherited in an autosomal dominant manner, but most individuals represent simplex cases (i.e., the only affected member in a family). It can find the RSTS individuals carrying pathogenic variation in a family with WES, but in the verification of parents by Sanger sequencing, parents generally do not carry the gene variation, indicating that this phenomenon is germline mosaicism or *de novo* variation and in the current state it is generally considered as *de novo* variation. In small families with only one child, the existence of germ cell mosaicism is not obvious. When the parents do not find the pathogenic variation, the variation is usually considered as *de novo*, but the possibility of germ cell mosaicism should be considered in genetic counseling, especially the possibility of father germ cell mosaicism. When the parents are clinically unaffected, their offspring and sib are still presumed to be at increased risk for RSTS because of the possibility of a mild phenotype in a heterozygous parent or parental somatic and/or germline mosaicism. The empiric recurrence risk for sib is less than 1% and the risk to offspring is 50%. Once the pathogenic variant has been identified in an affected family member, it is necessary that prenatal testing is taken for a pregnancy at increased risk and preimplantation genetic diagnosis is possible.

The RSTS has been subdivided into type 1 associated with the spectrum of *CREBBP* variant (RSTS1; NM_004380.3) and type 2 associated with the spectrum of *EP300* variant (RSTS2; NM_001429.4). Some studies suggested that RSTS patients carrying the *EP300* gene variant have a mild clinical phenotype [19], but Fergelot et al. previously conducted a comparative study on 52 children carrying the variants in the *EP300* gene and 308 children carrying the variants in *CREBBP* gene and found that the clinical manifestations of the two types were similar [6]. Some other studies also found more severe microcephaly and facial bone structure deformities in children with the variants in the *EP300* gene [12]. Our research team previously reported a RSTS patient with *CREBBP* gene variant, who had more severe clinical phenotype as compared with the proband in this study and facial deformity, significantly delayed language development, inability to communicate normally, and severe mental deficiency (IQ values of 10–20) by which RSTS is characterized [20].

In Asian populations, all reported RSTS patients have varying degrees of mental deficiency, and more than 90% of them have obesity, microcephaly, broad thumbs and/or hallux valgus, and 77% of them have combined ocular abnormalities, mainly strabismus [21]. Previous studies have also reported other serious ocular abnormalities in RSTS patients, including corneal abnormalities (19.7%, such as giant corneas without glaucoma, corneal opacities, and spherical corneas), congenital glaucoma (26.5%), congenital cataracts (12.8%), corneal defects (33.3%), and Duane’s syndrome (8%). The characteristic appearance of the eye includes ptosis, downward sloping palpebral fissures, ocular hypertelorism, and epicanthus [10]. In this study, the proband was complicated with early-onset high myopia (eoHM), in addition to esotropia and down-slanting palpebral fissures, which was rarely reported in RSTS patients. High myopia is defined as refractive error ≤-6.0 D or axial length > 26 mm [22]. According to the age of onset, high myopia is divided into late-onset high myopia (loHM), which occurs after school age, and early-onset high myopia (eoHM), which occurs before school age. Numerous genetic studies have shown that eoHM differs from loHM. EoHM, occurring before school age, is considered to be predominantly determined by genetic factors with minimal environmental effects(e.g., such as near work). eoHM can be classified as a simple type (non-syndromic) with high myopia alone and as a syndromic type with complicated with other eye diseases or systemic abnormalities, and so associated with the responsible genes. eoHM is closely associated with some genetic disorders and is often the main reason for the first diagnosis of some patients and the first clinical feature to attract the attention of clinicians [23–25]. The lack of awareness and attention given to this category of genetic disorders by ophthalmologists can easily lead to misdiagnosis or underdiagnosis. In this study, the patient was diagnosed with high myopia at the time of his first visit to the ophthalmology department at the age of 5, and due to mild systemic clinical manifestation, he was only treated with glasses for refractive error without further examination and genetic testing, leading to long-term misdiagnosis. It is suggested that eoHM may be the main reason for the earliest consultation in children and provide an important clue for genetic screening and further specific clinical examination for clinicians to discover underlying ocular diseases or systemic diseases. Therefore, in addition to the detailed examination of ocular structure and function in eoHM patients, high priority should be given to genetic screening to identify the pathogenic genes that can contribute to the early diagnosis, prompt treatment, and long-term follow-up assessment of some diseases.

The nonsense variation c.3714_3715del (p.Leu1239Glyfs*3) identified in our case could generate a PTC in *EP300* gene, which might be recognized and degraded by NMD. NMD is an mRNA quality monitoring mechanism widely found in eukaryotic cells. It can recognize the mutant mRNAs carrying PTCs and rapidly degrade and eliminate aberrant transcripts to prevent the accumulation of truncated and potentially harmful proteins by preventing the translation of the aberrant mRNA, thereby modifying the phenotypes. This efficiency is governed by several rules. The 50nt rule and the last exon rule are taken as canonical rules [26–28]. PTCs of less than 50 nucleotides (nt) upstream of the last exon-exon junction will trigger NMD (the 50nt rule), and PTCs in the last exon of a gene do not trigger NMD (the last exon rule). When it is predicted that the nonsense variant occurs at the 5′end of the same gene (usually greater than 50-55nt), it will trigger NMD to effect a decrease in mRNA expression, resulting in an insufficient synthesis of the corresponding proteins to perform normal physiological functions and thus cause disease. The NMD mechanism enables most mutated transcripts to be eliminated before translation, and transcripts carrying such PTCs have significantly lower levels of mRNA transcripts than wild-type proteins, and symptoms are usually less severe compared to those caused by PTCs at 3′end. If the nonsense variant occurs at the 3′end of the same gene and it is predicted that its termination codon is present in the last exon, or the last 50 base pairs of the penultimate exon, the nonsense variant can escape the NMD mechanism and nonsense variant-mediated transcriptional decay may not occur, and transcripts carrying such PTCs are transcribed at levels comparable to those of wild-type protein mRNAs, resulting in deleterious truncated proteins that lead to disease, often with a more severe clinical phenotype than that caused by PTCs at 5′end. Our study involved the variation, c.3714_3715del (p.Leu1239Glyfs*3), located on exon 21 in *EP300* (The *EP300* gene has 31 exons). As is consistent with the existing 50nt rule, the physical sites of this variant were not on the last or the penultimate exon as NMD is believed to play a role in the phenotypes. NMD can decrease its mRNA expression, resulting in an insufficient synthesis of the corresponding proteins to perform normal physiological functions and thus cause disease. Therefore, we used RT-qPCR to detect the expression of *EP300* gene transcript levels, i.e. relative expression of mRNA, in the proband and their normal family members. The results showed that the mRNA expression of *EP300* gene available with the samples I-1(1.04)、I-2(0.69), II-2(0.97)of the proband (II-1) (0.49) was lower than that in the samples I-1 (1.04), I-2 (0.69), and II-2 (0.97) of the normal family members without this nonsense variant in *EP300* gene indicating that most of the mutated transcripts were eliminated before translation due to NMD. It is not difficult to understand why the patients in this study had a mild clinical manifestation. The proband in this study had all of the typical features of RSTS as described above, namely obesity, microcephaly, and broad thumbs, however, the height of the patient was consistent with the development status of his peers, with mild mental retardation, certain language communication ability, and mild systemic clinical manifestation.

In addition to the typical systemic abnormalities of various organs in RSTS patients, previous reports have shown that benign tumors are more common in RSTS patients than in the general population, and a tendency to scar diathesis has been also described in RSTS individuals [29]. Many RSTS children will undergo multiple surgeries, and delayed recovery from anesthesia has been previously reported in RSTS patients, necessitating that anesthesiologists be alerted to the possibility of delayed recovery and use sedation medications with caution [30]. This shows that early diagnosis is particularly important in RSTS patients, allowing for comprehensive management and individualized treatment plans. Proper genetic counseling for the affected family is essential in the case of rare genetic diseases. Furthermore, parenteral genetic screening/diagnosis is the best strategy for managing this disease, which currently has no therapy [31]. For RSTS families, especially in highly consanguineous marriage populations, performing prenatal testing is necessary. Preimplantation genetic testing (PGT) can be performed for monogenic disorders or single gene defects (PGT-M) [32]. Also, cell-free fetal DNA (cffDNA) in the mother’s plasma has been introduced for autosomal dominant and autosomal recessive disorders [32]. Noninvasive and effective prenatal screening methods can help more families with rare genetic diseases conceive healthy children. When the genetic test results of the diseases indicate the uncertain significance of the variants, efforts should be made to distinguish the diseases as pathogenic or benign based on the physical examination, laboratory examination, and imaging data of the patients as the final clinical decision.

## Conclusion

RSTS is a genetic disease involving multiple organs with high genetic heterogeneity and diverse clinical phenotypes. The heredity and harmfulness of RSTS are serious enough to warrant prompt genetic counseling and prenatal diagnosis. Here, in this paper, we first report *de novo* heterozygous variation in *EP300* causing eoHM-RSTS. Our study extends the genotypic spectrums for *EP300*-RSTS and better assists ophthalmologists in predicting, and diagnosis.

## Data Availability

The datasets generated and analyzed during the current study are available in the [GenBank] repository (GenBank (https://www.ncbi.nlm.nih.gov/WebSub/) ID: 2,659,337).
